# Dusty Plasma Studies in the Gaseous Electronics Conference Reference Cell

**DOI:** 10.6028/jres.100.034

**Published:** 1995

**Authors:** H. M. Anderson, S. B. Radovanov

**Affiliations:** Department of Chemical and Nuclear Engineering, The University of New Mexico, Albuquerque, NM 87131

**Keywords:** Coulomb solids, discharge, dynamic laser light scattering, Gaseous Electronics Conference Reference Cell, laser light scattering, plasma processing, particles, particle traps

## Abstract

Particle “dust” in processing plasmas is of critical concern to the semiconductor industry because of the threat particles pose to device yield. A number of important investigations into the formation, growth, charging, transport and consequences of particulate dust in plasmas have been made using the Gaseous Electronics Conference Reference Cell as the reactor test-bed. The greatest amount of work to date has been directed toward a better understanding of the role that electrostatic, ion drag, neutral fluid drag and gravitational forces play in governing the dynamic behavior of particle cloud motion. This has been accomplished by using laser light scattering (LLS) techniques to track the motion of suspended particle clouds in rf discharges. Also, statistical correlation’s in the fluctuation of scattered laser light intensity [dynamic laser light scattering (DLLS)] can be used to determine information about particle size, motion, and growth dynamics. These results are reviewed, along with recent work demonstrating that charged dust particles in a plasma can form a strongly coupled Coulomb liquid or solid. New results from DLSS experiments performed in the Reference Cell are presented that show process-induced dust particles confined in an electrostatic trap exhibit low-frequency oscillatory motion consistent with charge density wave (CDW) motion predicted for strongly coupled Coulomb liquids.

## 1. Introduction

In the last 5 years, studies of particles in plasmas have focused on the impact of particles on the manufacture of microelectronic devices. Plasma etching and deposition are essential processing steps in the fabrication of microprocessors, memory chips and other devices with fine line features. The presence of particles in the plasma not only represents a potential contamination threat to processed wafers (and therefore manufacturing yield), but also, they can cause local perturbations in the plasma characteristics which adversely effect processing. The formation of particulate nuclei in a plasma, their growth in diameter from a few nanometers to several microns, the charging of particles in a plasma, the transport of these suspended particles and the consequences of particulates at the end of processing are not well understood.

The intent of the Gaseous Electronics Conference (GEC) Reference Cell was to provide a standard or common geometry for the study of laboratory rf discharges, with the hope that a standardized design would allow for direct comparison of experimental measurements between laboratories studying such complex phenomena as dusty plasmas. Furthermore, the characteristics of the Reference Cell design were selected so that the Cell had many features in common with commercial tools (showerhead feed gas inlets, reactive ion etch (RIE) mode of operation, etc.), while maintaining easy optical access to the discharge through multiple viewing ports. Since experimental detection of particles in plasmas is primarily accomplished through the use of laser light scattering techniques, the optical access afforded by the Reference Cell design is absolutely critical to the study of dusty plasmas. At the same time, since the Cell is to some extent similar to commercial tools, the knowledge gained from dusty plasma studies in the Reference Cell might be transferred more easily to improved commercial tool design.

**Fig. 1:**
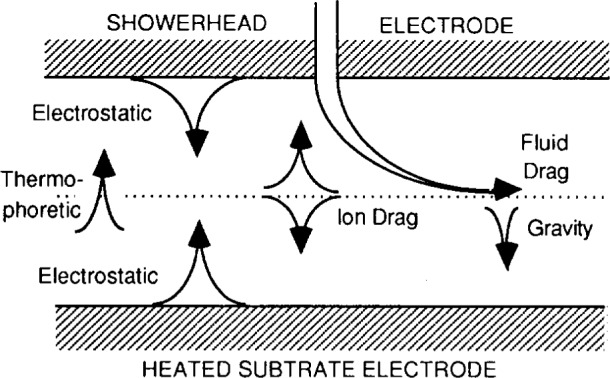
Forces acting on dust particles in a plasma processing tool (adapted from Choi and Kushner [1]).

Figure 1 graphically illustrates what is now believed to account for the balance of forces which dictate particle motion for dust particles suspended in the planar diode configuration of the Reference Cell, or for that matter, any similar planar diode rf plasma processing tool [1]. The position of the particle at any one time (and hence our ability to detect dust particles in plasmas) depends on the strength of electrostatic forces tending to confine or trap the particle in the discharge versus the fluid drag forces tending to sweep the particles out of the discharge. Gravity, ion drag, and thermophoretic forces (if electrode heating is present) can also affect particle motion to a greater or lesser extent depending on the size and mass of the dust. At the time the Reference Cell design was being established (1988–89), the level of knowledge with respect to particulate transport in plasmas was not well advanced. Little if any thought was given to particle trapping features of the Reference Cell design. The first dusty plasma studies in the reference cell [2] in fact gave the impression there was no particle trapping with the Reference Cell in the standard configuration. However, further work [3–6] has shown that modest modifications to the Reference Cell can and does lead to significant particle trapping in the gap region of the discharge.

The significance of the Reference Cell dusty plasma studies to date are largely concerned with modifications to the original design that cause the appearance of visible dust particle trapping. Some studies have shown that even simple modifications to the Reference Cell standard design (the introduction of a dielectric guard ring or a modification in the feed gas injection method) cause particle trapping. These effects are of significant interest to tool designers who routinely incorporate these features into commercial plasma reactors. Other, more radical modifications such as removal of the upper showerhead electrode altogether or elimination of gas flow are a significant deviation in the Reference Cell design concept, but they have lead to an improvement in our understanding of the physics of dust particle/plasma interactions. This paper will address the modifications to the Reference Cell design which lead to these observations and laser light scattering measurements which are essential to particle detection.

## 2. Optical Characterization of Particle Traps

When particles are either formed in a discharge due to process-induced plasma chemistry or intentionally injected into a discharge, experimental studies have shown that the particles rapidly acquire a net negative charge by electron capture (tens to thousands of charges) and accumulate in a specific region of an rf discharge, often the plasma-sheath boundary. Dust particles, slow and massive in comparison to elementary point charges, respond to the time-averaged electric field structure of the rf discharge and are repelled from sheath regions. Whether or not these particles remain trapped or suspended in the discharge by electrostatic forces depends on subtle details of the reactor geometry and process parameters. These details can cause variations in the plasma potential at sheath boundaries, the magnitude of the viscous ion drag (which accelerates particles in the direction of the net ion flux), the magnitude of viscous fluid drag forces, and even the mass of a growing particle.

Laser light scattering (LLS) has been the primary means of detecting particles [7]. In this technique, laser light is directed into the discharge parallel to the powered electrode at varying heights above the plasma sheath boundary. Usually the laser operates in the visible region as does the detector (photomultiplier or CCD array) which captures the scattered incident light at some angle to the laser beam. In general, light scattering depends on particle size, shape, index of refraction and the wavelength of the incident laser light. Particle sizing can be accomplished by taking into consideration the angular distribution of light scattering. For particles >0.5 μm and for visible light, Mie scattering occurs with the scattering component oriented primarily in the forward direction within a cone of about 15° acceptance angle. For particles between 0.5 μm and 0.2 μm diameter, transitional Rayleigh-Gans scattering occurs which progressively becomes more isotropic in nature. Below 0.2 μm diameter, Rayleigh scattering is dominant with the angular dependence of scattering disappearing altogether below about 40 nm. Some studies [7] raster the incident light with a rapid scanning mirror and use a video camera/lens assembly to capture a frame-by-frame record of the dynamic particle response to changes in the force balance on the particle.

Dynamic laser light scattering (DLLS), which measures fluctuations in the scattered light intensity, is especially useful for particle size and velocity measurement when particle diameters are below 0.2 μm [8,9]. DLSS does not depend on refractive index and it is not necessary to know the optical characteristics of such small particles. In the classical DLSS experiment, the incident light is a plane polarized electromagnetic wave:

$$E_{i}\;(r,t)=n_{i}\;E_{o}\;\exp\;i\;[k_{i}r-\omega_{i}t]$$ (1)

where ***n***_i_ is a unit vector in the direction ***r*** of the incident electric field, *E*_o_ is the field amplitude at time *t* and ***k***_i_ is a propagation vector, ***k***_i_ = (*ω*_i_/*c*) *k*_u_, when *k*_u_ is a unit vector specifying the direction of the propagation and *ω*_i_ is angular frequency. The scattered light field at the detector at any given time will be the superposition of the electric fields radiated from the particles in the illuminated volume. In the presence of changes in the particle position and orientation, fluctuations in the intensity of scattered laser light will occur which can be related to particle velocity and an average radius of gyration of the particle.

In the homodyne photon correlation spectroscopy version of DLSS, only scattered light impinges on the detector, so the instantaneous current at the detector is proportional to the square of the incident electric field:

$$\langle I(q)\rangle =\langle {\left|E_{s}\;(q,t)\right|}^{2}\rangle$$ (2)

where *q* = (4π/λ) sin *Θ*/2 is a scattering wave vector. Therefore, the amplitude of the scattered field is proportional to the number of emitted photons, which is time dependent. This method yields the time autocorrelation function which can be defined as:

$$\langle I(0)\;I(\tau )\rangle =\lim\;1/T\;\int \;I(t)\;I(t+\tau^{\prime })dt$$ (3)

where *t* is time, *τ*′ is the shift or delay time, and *T* is the integration time over which the measurement is averaged. This time autocorrelation function of the number of photons arriving at the detector with a high speed photomultiplier and digital signal processor can be approximated by a finite sum of *N* products. This sum is obtained by sampling the signal in discrete intervals of equal duration:

$$f(j\Delta t)=\lim\;(1/N)\;\Sigma \;n_{i}n_{i-j}$$ (4)

for *j* = 1,2,3,…, *M*. Here *n_i_* and *n_i_*_−_*_j_* are the current number of pulses that are multiplied by the contents of the *j*th channel and *M* is the total number of channels in the correlator.

The time autocorrelation function decays in the time, *τ*, a typical particle moves through a distance *r*. In most applications of DLSS, this decay has two characteristic shapes [9]. If the mean particle motion dominates (i.e., there is only Brownian or random thermal motion), the shape of the decay is Gaussian and the mean velocity of the particles can be evaluated from the decay time and the scattering wave vector, i.e., 〈*u*〉 = (*τq*)^−1^. If the particles have little or no mean velocity compared to the convective velocity (i.e., hydrodynamic motion), the shape of the decay is Lorentzian and the decay time can be used to calculate the particle transit time and a friction coefficient. Hurd and Ho [8] have established that in the specific case of submicron charged particles in the rarefied atmosphere of a glow discharge plasma, the decay function shares elements of both these motions. Despite the complex shape of the decay function in this instance, they show it is possible to acquire a reasonably accurate estimate of a plasma dust particle mass and size by fitting the initial portion of the autocorrelation curve with a Gaussian function. The velocity associated with the decay time *t* of this Gaussian fit is related to the particle mass by 〈*u*〉^2^ = *kT*_p_/*m*_p_, where *m*_p_ and *T*_p_ are the mass and temperature of the particle. Assuming that the particle is roughly spherical and an approximate density is known, the particle radius of gyration can be calculated. The radius of gyration can be related to the particle radius dependent on whether the particle is a single sphere or a composite of agglomerated spheres.

The above analysis does not take into account the rf driven motion of the charged particles or other forces acting perpendicular to the electrode surface (electrostatic, ion drag, thermophoretic or gravity) in a parallel plate reactor. It presumes particle motion in the radial direction of a parallel plate reactor occurs either through random thermal motion or convective flow. It is important to note that this presumption is reasonable for very small particles formed shortly after igniting a discharge by process-induced chemistry. Small particles in a discharge will be only weakly charged or possibly neutral, and their motion will be largely unaffected by electrostatic particle traps until they drift substantial distances parallel to the electrode surface and encounter retarding traps. We will discuss in much greater detail the potential impact of electrostatic and ion drag forces on the shape of the autocorrelation function later in this report, when new DLSS data is introduced on small particle motion in a discharge.

## 3. Summary of Dusty Plasma Reference Cell Modifications and Experiments

The initial attempts to study particle behavior in the Reference Cell were a profound disappointment simply because particle trapping did not appear to occur to any significant extent when the Reference Cell was in the standard configuration (showerhead upper electrode and lower electrode present) under typical flow/power/pressure conditions. Selwyn [2] observed that under these conditions, particles introduced into the discharge where swept out of the gap region by fluid flow and were only weakly trapped at the base of the lower electrode. In such a location, the viewing angle on the particles illuminated by laser light scattering is quite poor, giving the impression no particle traps exist.

**Fig. 2:**
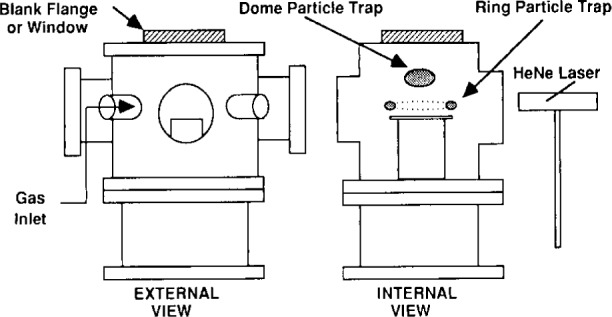
Generic schematic of modification to Reference Cell showing the general configuration adopted by Selwyn’s group [3], O’Hanlon et al. [5] and Goree’s group [6]. Each group has slight variations on this design.

Selwyn first suggested a modified version of the Reference Cell would be more appropriate for particle transport studies. This modification [3] involved removing the upper electrode (including the ground shield) and introducing gas flow to the chamber via a single side mounted gas injection port, as illustrated in Fig. 2. In this configuration, 30 W of rf power at 13.56 MHz is capacitively coupled to the lower electrode. Due to the even more pronounced asymmetric nature of the reactor configuration, a substantial dc self-bias develops at the powered electrode, measured to be −310 V at 6.65 Pa (50 mTorr). A typical flow rate of 37 μmol/s (50 sccm) Ar is used in experiments described to date. O’Hanlon, Kang, and Russel [5] have also investigated the influence of the conventional Reference Cell configuration versus running the cell with the top electrode removed. Both groups have reported seeing the appearance of a large ring trap surrounding the powered electrode, as well as a dome of particles over the electrode when the Cell was modified.

Recent experiments of Dalvie, Surrendra, Selwyn, and Guarnieri [3] have focused primarily on particle trapping and transport issues, as opposed to plasma chemistry, particle generation, and growth. For this reason, particles were introduced to the chamber by means of a filtered particle generator mounted over the electrode. The advantage of such a scheme is that particle sizes can be closely controlled, as opposed to having a distribution of particle sizes which would result from any spontaneous nucleation process. The primary focus of this work was to investigate the impact of millimeter scale electrode topographic features on the formation of electrostatic particle traps in the discharge. A 10 cm×10 cm Al plate was used in place of the typical circular lower electrode. The Al plate had a 22 mm×6 mm groove down the centerline, so as to intentionally introduce a particle trap in the equipment. Spatially resolved optical emission spectroscopy (OES) measurements were used in conjunction with conventional LLS measurements to determine the correlation between Ar excitation (420.1 nm) spatial variation and the appearance of well defined particle traps associated with the groove.

The results of this study show a clear correlation between bends in the contour lines of Ar 420.1 nm emission and the formation of a local plasma potential well which serves as an electrostatic particle trap. The groove is shown to induce an enhancement of the local ionization rate near the side wall as the result of extra electron heating near the sharp edge at the top of the groove. Although it is not entirely clear as to whether there is a direct correlation between the enhanced emission and ionization rates associated with the groove perturbation, both events are linked to perturbations in the plasma potential which result in electrostatic particle trapping. Selwyn et al. [7,10,11] have also reported many other electrostatic trapping phenomena associated with various aspects of tool design such as clamp rings, electrostatic chucks, and other features which introduce topographic variation. However, most of these other studies appear to have been performed in tools other than the Reference Cell and its characteristic single wafer processing configuration.

**Fig. 3:**
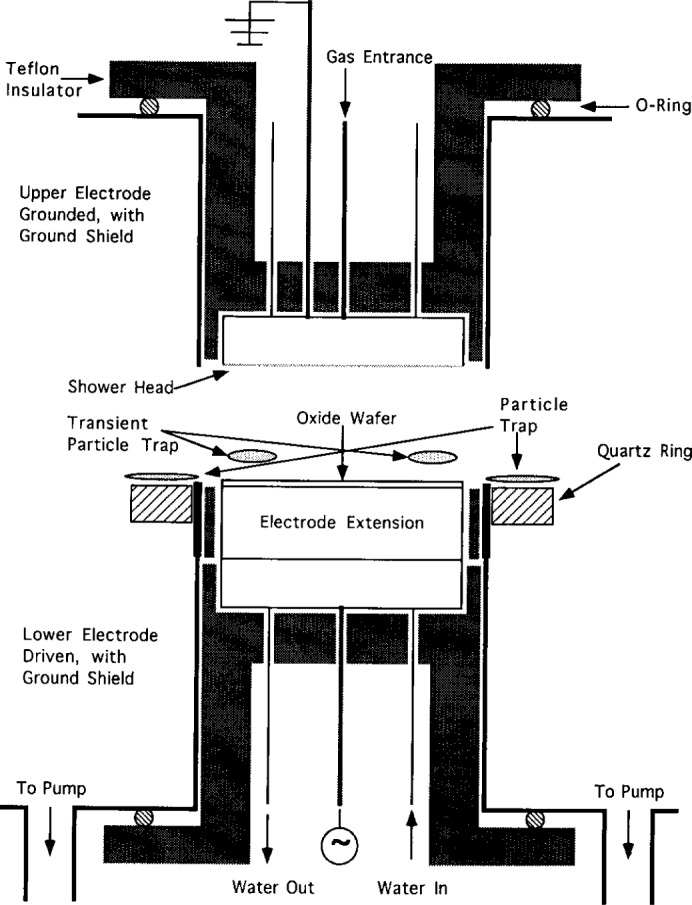
Confinement ring modification to Reference Cell by Anderson et al. [4].

**Fig. 4:**
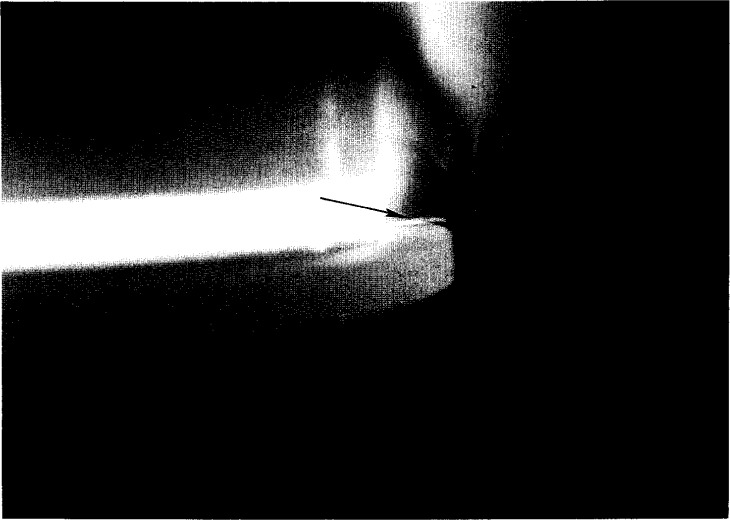
LLS image of particle trapping in confinement ring modified Reference Cell. The arrow points to the illuminated portion of the ring particle trap. The ring particle trap encompasses the entire circumference of the electrode.

Anderson et al. [4] have taken a slightly different approach in work that has focused much more extensively on the role of processed-induced particle formation, the role of plasma chemistry in particulate nucleation and DLSS measurements in the Reference Cell. Their only modification of the Reference Cell standard design is a quartz guard ring around the lower powered electrode. A schematic of this modification to the Reference Cell and the resultant particle traps are shown in Fig. 3. A photograph of trapped, suspended particles is shown in Fig. 4. Confinement rings and/or clamps around the periphery of the electrode are in common use in commercial reactors. The addition of the quartz confinement ring to the Reference Cell electrode appears to cause a major change in the plasma potential distribution at the radial edge of the electrode and the consequent formation of a particle trap. Most likely this is due to the dielectric discontinuity of a quartz ring adjacent the Al metal electrode. The ring was mounted level with the electrode surface in order to minimize any disruptions in the viscous fluid flow pattern in the reactor.

Anderson et al. [4] have used this configuration to perform an exhaustive study on the role of etch products in particulate generation during the oxide etch process. For these studies, a blanket oxide wafer was present on the powered lower electrode (i.e., the typical single wafer RIE mode of etching). These studies were performed using a typical oxide etch chemistry of CHF_3_/CF_4_ with the CHF_3_ composition varied between 20 % to 50 %. Pressure was held constant at 93.1 Pa (700 mTorr) and flow was varied between 7.5 μmol/s to 29.8 μmol/s (10 sccm to 40 sccm). Although qualitative, the intensity of the LLS signal in these studies appeared to be highly correlated with the progress of the oxide etch, indicating that the Si and O etch products play a significant role in particulate formation. Since light scattering persisted well after the oxide film had been etched, it was apparent that fluorocarbon fragments such as CF_2_ can also interact to form particulates (presumably polymeric), but at a much slower rate than when the plasma chemistry is enriched with wafer etch products. This study also demonstrated that the morphology of particulates formed in the discharge changed significantly under very high power, high field conditions in the sheath region. Particles which normally appeared as a complex agglomerate of ~200 nm diameter spherical primary particles became filamentous, string-like, fused-particle chains under high field conditions. This type of agglomeration is known as field-aligned coagulation and is a well known phenomena in the aerosol sciences.

Experiments by Cui, Goree, and Quinn [6] with a modified Reference Cell (upper electrode removed) have been recently reported in the 47th Annual Gaseous Electronics Conference. These studies have focused on phase transitions of plasma particle clouds to macroscopic crystalline structures. They refer to these crystalline structures as Coulomb crystals or as a “plasma crystal.” The transition from essentially random aggregates of dust to a structure with more long range order requires special conditions described by a Coulomb coupling parameter:

$$\Gamma ={Q_{p}}^{2}/4\pi e_{o}kT_{p}\Delta$$ (5)

which is the ratio of the interparticle Coulomb potential energy to the thermal energy. In Eq. (5), *Q*_p_ is the particle charge, *T*_p_ is the particle temperature, and *Δ* = (3/4π*n*_p_)^1/3^ is the interparticle distance for a particle density of *n*_p_. For *Γ* ≥ 178, a Coulomb solid forms.

**Fig. 5:**
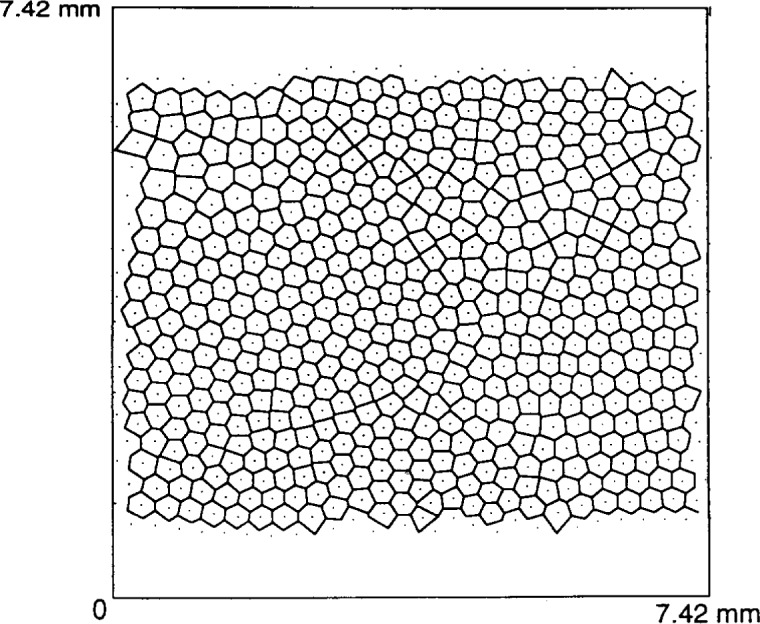
Voroni (Weigner-Seitz) Cells observed by Goree’s group [6].

In these studies, the fluid drag force is entirely eliminated by removing flow to the reactor. Thermophoretic forces are also negligible. This reduces the force balance acting on particles to electrostatic, ion drag and gravity components. These Reference Cell studies were performed in a krypton discharge at low rf powers and 66.6 Pa to 133 Pa (0.5 Torr to 1 Torr) pressures. Pre-formed particles in the size range of 3 μm to 9 μm were injected into the discharge via a dust dispenser. The particles were seen as a levitated, thin, disk-shaped cloud near the sheath plasma boundary at the lower, powered electrode. Images of this cloud established the presence of regular Voronoi (Wigner-Seitz) cells in the plane of the cloud. This type of cell formation is illustrated in Fig. 5. If the rf power is raised, the particles move more violently, causing the breakdown of the solid crystalline structure. The results observed in the Reference Cell have not yet appeared in regular journal publications, but similar work has been published work on Coulomb solid formation in another test cell [14].

The work on Coulomb solids in dusty plasmas is of significant fundamental interest to the study of phase transitions, but it is unclear what significance it holds for plasma processing. Recent results from a series of dynamic laser light scattering experiments performed on the University of New Mexico GEC Reference Cell provide some interesting new insight into this question.

## 4. Dynamic Laser Light Scattering in the UNM Reference Cell

This study was conducted in order to extract information on the dynamic evolution of particle size, speed and morphology during an oxide wafer etch cycle and to study collective particle behavior in an electrostatic trap. Particles observed in the Cell formed spontaneously by process-induced plasma chemistry, grew over time, and migrated radially in the discharge toward a ring particle trap by both random thermal motion and convective flow. There were sufficient particles formed in the first second of the etch process to allow detection by DLLS near the sheath boundary over an oxide wafer, even though they were not apparent to the naked eye. There did not appear to be any permanent localized electrostatic trap regions over the wafer with sufficient strength to confine these particles for any length of time. These particles obviously grew very rapidly and became entrapped in the convective flow stream since particles appeared, visible to the eye, in an electrostatic trap at the electrode edge within 3 s to 5 s. These larger particles, visible by LLS are shown in Fig. 4.

In this report, we will present DLLS measurements made at very short times (~1 s) and longer times (~3 s and 25 s) in the etch process. These snapshots in time of the fluctuations in particle light scattering intensity capture information on particle speed and size at various stages of growth of the rapidly evolving particle. The information is contained in the shape and decay of the autocorrelation function. Rapid particle growth, in addition to thermal motion and convective flow, becomes a factor which influences the shape of the autocorrelation function when trying to obtain information about particles in a glow discharge at short times. This additional factor adds to the complexity of what is contained in the autocorrelation function, convolved with the previously noted kinetic to hydrodynamic “crossover” behavior in the motion of submicron particulates in a discharge. Furthermore, it will be shown that complex plasma oscillatory behavior can complicate the matter even further.

**Fig. 6:**
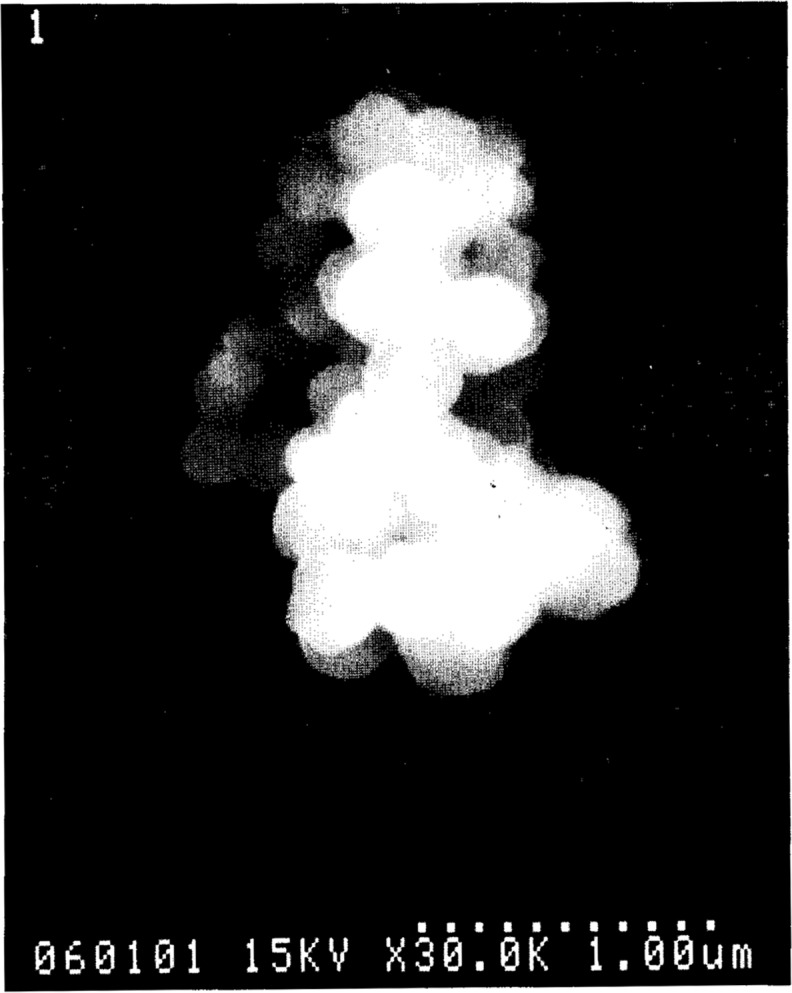
A scanning electron microscope micrograph of an agglomerated particle wafer contaminate produced at low power in the Reference Cell.

Process-induced particles often grow by a cluster interaction or coagulation process [15], followed by a more open agglomeration of primary particles [16]. Coagulation and agglomeration are the processes which make possible the very rapid growth of particles to sizes visible by LLS noted in this study. The typical final particulate product in a fluorocarbon etching discharge is illustrated in Fig. 6. This micrograph of a typical particle contaminant found as residue on a wafer after etching is composed of primary particles ~200 nm in diameter. These primary particles form a larger agglomerate on the order of 1 mm diameter. We will discuss aspects of the nucleation and early growth of primary particles in the context of DLLS measurements made in the initial second of the discharge in Sec. 4.2. In Sec. 4.3, we will discuss the collective behavior of many larger, trapped agglomerated particles in the context of DLLS measurements made at later times in the discharge.

### 4.1 Experimental

The Cell was configured with a powered lower electrode, a shower head gas inlet on the upper electrode, and a Teflon insulator. The gap space was reduced to ~1 cm by adding an aluminum electrode extension to the standard electrode. A quartz guard ring, 1.27 cm thick, was machined to slip around the powered electrode, level with the plane of the electrode surface (see Fig. 3). The Cell was operated at a constant pressure of 93.1 Pa (0.7 Torr) in two regimes of the parameter space of a CF_4_/CHF_3_ discharge: high power (350 W)—low flow (10 sccm) and low power (90 W)—medium flow (40 sccm). Typical peak-to-peak rf applied voltages in the Reference Cell for the high power case correspond to ~1250 V, whereas for low power, the peak-to-peak rf applied voltages are ~500 V. The volume ratio of CHF_3_ to CF_4_ in the cell was maintained at a constant value (1:1) for the DLLS experiments. Several 100 mm blanket, n-type thermal oxide wafer (1000 nm thick) were used as substrates. This choice of conditions in the Reference Cell operational parameter space was made in order to sample extremes of the parameter space where particle formation is known to occur in commercial processing tools.

A Coherent^1^ DPSS 532 series solid-state, diode-pumped Nd:YAG frequency doubled laser was used to produce light at 532 nm wavelength, while a Brookhaven model BI 2030 AT digital, high speed photomultiplier/signal processor was used to perform the photon correlation spectroscopy on the scattered light signal. In the DLLS experiments, vertically polarized light was focused 1 mm above the quartz guard ring. Scattered light was collected from two locations, one being roughly 10 mm in from the edge of the powered electrode (identified as the transient region in Fig. 3) and the second being at 1 mm out from the edge of the powered electrode (identified as the particle trap in Fig. 3). Scattered light from the location over the wafer was collected under the high power reactor conditions. Scattered light from the location at the wafer edge was collected under the low power reactor conditions. The measurement in each location was repeated several times. The light scattered from the particles was collected with a 50 cm focal length lens in individual runs at horizontal scattering angles from *θ* = 3° to 20°. The scattering wave vector was parallel to the electrodes so that the rf driven particle motion would not affect the measurements. The scattered light was focused in a 200 μm masking pinhole to reject flare light and then passed to a photomultiplier tube. Prior to the DLLS discharge measurements in the Reference Cell, the DLLS system was calibrated using pre-formed 1 μm size particles in an atomizer cell.

### 4.2 DLLS Measurements and Early Particle Growth

Light scattering was observed within a second of striking the discharge from the region over the wafer. However, this initial DLLS signal was obscured within a few seconds because of intense scattering from particles trapped at the edge of the electrode. The intense scattering from particles at the wafer edge persisted into the later stages of the etch, preventing further optical access to the region over the wafer at later times.

**Fig. 7:**
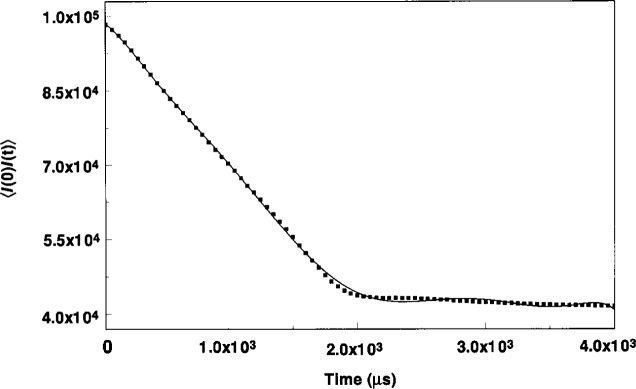
High power condition autocorrelation function obtained by DLSS from Reference Cell over the wafer at one second after initiating the discharge.

In the early stage of particle formation, the particles were small and quite mobile. Figure 7 shows a time autocorrelation function typical of the fluctuating light scattering occurring over the wafer under the high power condition. This measurement began approximately in the first second after initiating the discharge. The shape of the decay in the correlation function appears at first sight to be nearly linear, but subsequent curve fitting analysis indicates the best fit is a linear plus Gaussian function shown by the solid line in Fig. 7. The near linear decay in the autocorrelation function can be explained by examining Eq. (3). If the decay time for scattered light fluctuations (due to small, fast moving particles) is shorter than the sample time used to generate the autocorrelation function, then the measured photon pulse *I*(*t* + *τ*) taken at the delay time *τ* will resemble the original photon pulse *I*(*t*), assuming the motion of particles is primarily random. Under these conditions, the time autocorrelation function 〈*I*(0) *I*(*τ*)〉 =lim 1/*T* ∫ *I*(*t*) *I*(*t* + *τ*) d*t* will yield a linear function since ∫ *I*(*t*) *I*(*t*) d*t* = 2 *f*(*I*(*t*),*t*)^2−1^ if *I*(*t* + *τ*)≈*I*(t). Pecora [17] notes that this can be of major importance for very dilute particle suspensions when there are few particles in the scattering volume.

Attempts to obtain a better resolution in the decay of the autocorrelation function by selecting a shorter sample time are incomplete at this time. Using the shortest sample time (1 μs) of the Brookhaven correlator resulted in a poor signal to noise ratio and poor reproducibility of the decay profile. Again, this indicates the concentration of particles in the scattering volume is small and the particle motion fast. The most that can be inferred from Fig. 7 is that the decay time *τ* for kinetic particle motion in the initial stage of growth is less than 50 μs. Using this value as an upper bound, we can estimate a root-mean-square velocity (*τq*)^−1^ of the particles as >2.8 cm/s. However, even this value is probably low. At the 10 sccm flow rate, the radial convective gas flow velocity in the Reference Cell is on the order of ~5 cm/s, and it is expected that the kinetic velocity of submicron plasma dust particles would be well in excess of that based on the kinetic-hydrodynamic observations of Hurd and Ho [8]. We are unable to resolve this issue further without improving the sample time limits of our detector and possibly the strength of the light scattering signal by using a higher power laser.

Although Fig. 7 cannot be used to specify a precise *τ* for the decay in the kinetic motion of the small initial particles, it does offer proof for the existence of very small particles in the first second of the discharge and does allow us to at least set some bounds on what those particle sizes might be. Using *τ* = 50 μs as the upper bound for the kinetic decay, the average mass of the particles can be estimated using 〈*u_z_*^2^〉 = *kT*/*m*. With an assumed particle density of ~2.0 g/cm^3^, and temperature of 310 K, the average radius of gyration can be estimated as no greater than 70 nm. In all likelihood the particle radius is much smaller than this, but even with this upper bound value, it must be assumed that the DLLS signal is equated with single primary particles at an early stage of growth.

Boufendi and Bouchoule [15] have shown that in the first second of striking an Ar/Si discharge, a large burst (1×10^9^/cm^3^) occurs in the concentration of 10 nm primary particles made of small silicon substructures. Therefore, the above estimate of initial particle sizes in a fluorocarbon etching discharge does not appear to be unreasonable, although probably high as noted. What is perhaps more surprising is that there are sufficient densities of these small particulates in an etching discharge (as opposed to a deposition discharge like Si) to generate any DLLS signal at all in the first second of etching. In the case of a fluorocarbon etching discharge without a silicon substrate, Buss and Hareland [12] have reported substantially longer first appearance times (11 s) for polymeric CF*_x_* particles. *With a wafer present* in the discharge in this study, the DLLS measurements suggest initial particle growth is more akin to that observed by Boufendi and Bouchoule and points strongly to silicon etch products from the wafer surface playing a dominant role in initiating particle growth. Whether these etch products are radical species or larger stable atomic clusters as has been suggested for etching discharges [4,13] is unclear. The DLLS measurements could be consistent with either mechanism.

### 4.3 DLLS Measurements and Particle Behavior in a Strong Electrostatic Trap

**Fig. 8:**
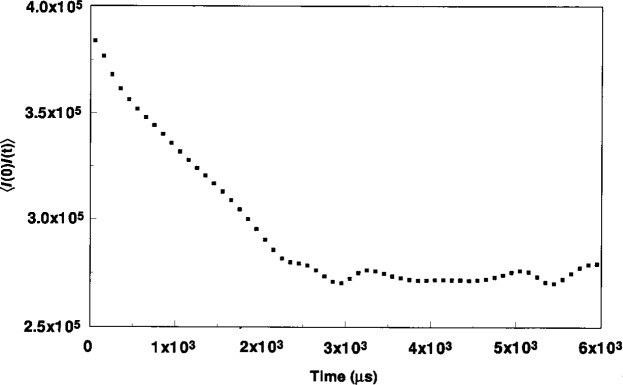
Low power condition autocorrelation function obtained by DLSS from Reference Cell in the ring particle trap at three seconds after initiating the discharge.

**Fig. 9:**
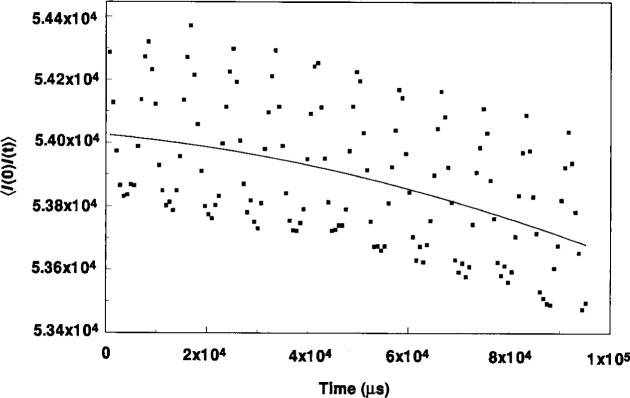
Low power condition autocorrelation function obtained by DLSS from Reference Cell in the ring particle trap at about 25 s after initiating the discharge.

In the later stages of the etch process, the particles were much more massive and visible to the eye by LLS at the radial edge of the discharge. DLLS measurements at the wafer edge could be made at any later time during the ~10 min etch cycle. After a lag time of about 10 s–15 s, there was little visual change in the LLS from the trap region. Figures 8 and 9 show the time autocorrelation function at two later times (~3 s and 25 s) into an etch cycle. Figure 8 was taken under the high power condition, Fig. 9 was under the low power operating condition. These signals correspond with the visible light scattering occurring over the confinement ring in Fig. 4. The intensity of LLS in Fig. 4 from the particle trap region shows the trap is densely populated with particles. Numerous pervious studies [10,16] have shown particle densities under such conditions reach 10^6^/cm^3^ to 10^8^/cm^3^.

The autocorrelation function taken at ~3 s into the etch and shown in Fig. 8 is quite complex. The overall shape of the correlation function has the same linear-like trend as that seen in Fig. 7 taken two seconds earlier over the wafer, with two notable exceptions. At the beginning of the sample period, there appears to be the beginning of a characteristic Gaussian profile, suggesting the primary particle has grown significantly in two seconds and the kinetic motion of the particle shows some sign of being resolved using a 50 μs sample time. At later times, there is a noticeable damped oscillation superimposed over the long term decay.

Despite the complexity of this autocorrelation function, its features can be explained in the context of events occurring in the particle trap. If the particles have grown to the 200 nm primary particle size, the decay time for kinetic motion would be on the order 60 μs, which appears consistent with the early Gaussian decay of the correlation function, even though the decay is not well resolved. This suggests the trap is beginning to fill with primary particles, a reasonable expectation at three seconds into the discharge. Choi et al. [18] have shown in model simulations of parallel-plate reactors that both ion drag and high gas flow can accelerate particles to velocities that are sufficient to overshoot the equilibrium position where electrostatic forces balance inertial forces. These particles then oscillate about the equilibrium position until fluid drag damps the oscillation. It is reasonable to anticipate that the primary particles filling the particle trap in this experiment are undergoing overshooting and oscillation within the trap in the Reference Cell, giving rise to the damped oscillations seen in Fig. 8. It is unlikely that ion drag contributes much to the overshoot since the particles are still small and therefore at a low charge state. However, the random thermal velocity of 200 nm particles (~2.2 cm/s) aided by the convective flow radial velocity (~5 cm/s) could cause overshoot.

Oscillations driven by this type of inertial force are random in nature, since particle generation and hence particle arrival times at the trap are variable. The oscillations in the autocorrelation function of Fig. 8 appear to be random, although at long times there is a slight hint of some regular oscillation. Since the trap must be rapidly filling due to the high flux of primary particles, it is impossible to say whether these oscillations are due to spurious variations in particle flux entering the trap or whether this is the beginning of some type of collective particle oscillation. What is most important to note is the oscillations in autocorrelation function decay appear only when viewing the trap region of the discharge at some latter time into the etch. These oscillations are real and not an artifact of noise pick-up from any extraneous electrical or stray light source.

Figure 9, taken ~25 s into the etch cycle, illustrates the autocorrelation function behavior when the trap is densely filled with particles. The most obvious feature is a well established regular oscillation, consistent with a collective linear harmonic motion of the particles in the trap. The frequency of this oscillation is between 120 Hz to 130 Hz. The oscillation is persistent, lasting over the 100 ms sampling duration, and in fact, much longer. DLLS measurements made many seconds later show the same characteristic oscillation at between 120 Hz to 130 Hz. The oscillation appears to be superimposed over a longer term Gaussian decay, indicative of the motion of a much larger, slower moving particulate structure. The solid black line represents a portion of a fitted Gaussian function to the longer term decay. The oscillation frequency does not change as a function of scattering angle. The wavelength of the oscillation, λ_L_, is ~1.4×10^−4^ cm and the phase velocity is ~1.75×10^−2^ cm/s. Linear wave theory analysis of the wave motion shows the propagation constant *k* ≅ 45,000 and the wave displacement or so-called linearized Debye length λ_LD_ ≅ 2.23×10^−5^ cm.

Using either 200 nm or 1 μm to 2 μm values for the diameter of particles filling the electrostatic trap, it can be shown that the experimentally observed oscillation frequency is consistent with a particle plasma oscillation frequency. Choi and Kushner [1] have produced models for the charge accumulated by a single particle as a function of size. These models show the charge scales roughly with size and plasma electron temperature. We expect the CF_4_/CHF_3_ discharge in the Reference Cell to be somewhat cool, on the order of perhaps 1 eV to 2 eV. Using this scaling factor, a reasonable estimate of *Q* on a 200 nm diameter particle is ~100 and on a 1 μm to 2 μm particle ~1000. The particle plasma oscillation frequency in Hz is given by *f*_p_ = (π*n*_p_ *Q*_p_^2^/*m*_p_)^1/2^ Since the charge on the particle scales with size, the particle density in the trap region necessary to have the particle oscillation frequency *f*_p_≅130 Hz is relatively insensitive to *n*_p_. For 200 nm particles, *n*_p_ must equal ~2×10^5^/cm^3^, whereas for 1.5 μm particles, *n*_p_ must equal ~6.7×10^5^/cm^3^. These values for particle densities in the trap are quite consistent with dusty plasmas observations and point to the fact that the observed experimental oscillation frequency is quite close to the plasma particle oscillation frequency of a dense dusty plasma.

Using these estimates for *Q* charges on the trapped particles and *n*_p_ particles in the trap, it is also possible to calculate both a theoretical interparticle spacing *Δ* and Coulomb coupling constant *Γ* for such a system of charged particles. The results are most interesting. For 200 nm diameter particles, the value of *Γ* is 0.4, indicating that if the trap was filled only with primary particles, the particles would not be strongly coupled or capable of supporting coupled harmonic motion. On the other hand, if the trap were filled with particles 1.5 μm in diameter, the value for *Γ* is ~60 and the particles would be expected to be strongly coupled. The evolution in the development of a regular, persistent oscillation captured in Figs. 8 and 9 is consistent with the particles in the trap undergoing such growth. Primary particles first caught in the trap would be subject to continuous bombardment from the flux of newly generated primary particles moving toward the trap, resulting in particle growth through agglomeration as suggested in Fig. 6. As such a particle grows in the trap, it would grow both in mass and charge to the point where strong coupling could occur.

The value of λ_LD_ is also consistent with strong Coulomb coupling between trapped particles, if the particle is a large agglomerate. Daugherty et al. [19] have shown that when the charged particle radius is on the order of λ_LD_ and larger, the system of charged particles have a Debye length on the order of the electron Debye length. For agglomerated particles of the size shown in Fig. 6, the particle radius, ~7.5×10^−5^ cm > λ_LD_ ≅ 2.23×10^−5^ cm and the condition is met. Assuming an electron temperature of ~2 eV and a density of ~1×10^9^/cm^3^, the electron Debye length for this fluorocarbon plasma is on the order 3×10^−2^ cm and there would be many charged particles within such a Debye sphere, satisfying the definition of a plasma.

Computer simulations of a classical one-component plasma (OCP) by Ichimaru [20], Hansen et al. [21] and others are also consistent with the type of oscillation shown in Fig. 9. Hansen has shown a charge-density-wave (CDW) instability arises in strongly coupled plasmas during the transition from the Coulomb liquid to Coulomb solid state. Using a velocity autocorrelation simulation, Hansen shows this oscillation begins to develop for values of *Γ* greater than one, and is quite pronounced for *Γ* = 50−150. The simulated behavior of the CDW is remarkably similar to that observed in the DLSS experiments above in that the simulated autocorrelation function has an oscillatory decay, superimposed over a longer time Gaussian decay function. The frequency of the simulated oscillation was nearly constant over a range of *Γ* and had a value 0.91 *ω*_p_, the particle plasma oscillation frequency in the OCP simulation. This is also consistent with our experimental oscillation frequency, as noted.

Chu et al. [22] and Stoffels et al. [23] have independently observed persistent oscillations of charged particles in dusty plasma glow discharges with strong Coulomb coupling. However, in both instances, the frequency of the oscillations was about an order of magnitude less than those observed in this study. In Chu’s study, the oscillations damped at pressures greater than 40 Pa (300 mTorr) and a Coulomb solid formed. In Stoffel’s study, the oscillations occurred only at low flows (<30 sccm). In both instances, these very low-frequency oscillations (~10 Hz) appeared to be related to charge fluctuations on single charged particles which caused collective density fluctuations. Stoffel characterized this oscillation as a plasma coughing, or a way for the particles to exhaust excess charge build-up. This phenomena may be relevant to the oscillations in Fig. 9, but the coughing in this study would be at a much higher rate.

These disparities (higher oscillation frequency and lack of wave damping at higher pressure or flow) can be accounted for if there is an entirely different pumping mechanism for charge fluctuations in the Reference Cell discharge. The fact that this oscillation is resistant to damping by viscous fluid flow may be due to the continuous flux or pumping action of new primary particles flowing into the trap region. Primary particles streaming across the bulk plasma/trap interface would be carrying information about their thermal velocities. If the propagation constant *k* for the wave is large (i.e. λ_LD_ is small), the wave should travel at thermal velocities [24], which it appears to do at ~1.75×10^−2^ cm/s, the magnitude of the thermal velocity of a 200 nm primary particle. This suggests the charge fluctuation that drives this oscillation is not charge fluctuation on a single particle, but rather total charge in the trap. From a simple charge balance over the trap, it can be seen that the additional charge added at the front of the trap by a continuous primary particle flux would have to be balanced by a charge exhaust at the back of the trap, otherwise the charge and mass would overwhelm the potential well of the trap. This is equivalent to the type of phenomena of plasma coughing as described by Stoffels et al. [23], but since it is driven by the dynamic production of new charged particles, it would be independent of pressure or flow.

In summary, we believe the most consistent explanation for the oscillations shown in Fig. 9 is as follows. When the electrostatic particle trap first fills with 200 nm particles, there is insufficient coupling between particles for harmonic oscillation to occur. However, the etch process and generation of new primary particles is ongoing and there will be a continuous flux of primary particles into the trap. The result appears to be particle growth by agglomeration with little change in total particle number density in the trap. Large particles in the trap are strongly coupled and they develop a CDW oscillation characteristic of Coulomb liquids. This instability does not damp because of the continued pumping of the plasma particle density wave by new charged particles streaming into the edge of the trap. This process continues until the source of process-induced particles is exhausted.

## 5. Conclusions

DLLS studies conducted in the Reference Cell reveal another instance where harmonic oscillations appear to develop in the motion of charged particles in a dusty plasma. A consistent explanation for the persistence of this oscillatory motion has been presented in the context of the charged dust particles forming a strongly coupled Coulomb liquid. There are of course many possible alternative mechanisms which could produce the appearance of oscillatory particle motion in a plasma, but it is difficult to reconcile these alternatives with either the time scales of the observed oscillation or its persistence.

The formation of a true Coulomb solid in the Reference Cell under any conditions with flowing gas is unlikely, but it is very interesting that strong trapping induced by seemingly benign modifications to the Reference Cell standard design appear to bring the trapped particles so close to the Coulomb liquid-solid phase transition. We believe this is the first experimental evidence linking charge density waves and Coulomb liquid-like behavior to charged dust particles in an etching plasma. The fact that the dust particles appear to exhibit such a high degree of collective behavior under actual processing plasma conditions has important commercial implications.

The research conducted on dusty plasmas in the GEC Reference Cell, including studies on particle formation, charging, transport and release, has been reviewed. Tantalizing new information about strong plasma particle coupling obtained by dynamic laser light scattering has been presented. These findings show that even with the standard Cell design relatively unchanged and under conditions that are very relevant to plasma processing, strong Coulomb coupling may be occurring. Further studies to better understand this effect on plasma processing are needed.

## Footnotes

1 Certain commercial equipment, instruments, or materials are identified in this paper to foster understanding. Such identification does not imply recommendation or endorsement by the National Institute of Standards and Technology, nor does it imply that the materials or equipment identified are necessarily the best available for the purpose.
